# The effects of intermittent fasting diet in comparison with low-calorie diet on lipid profile, glycemic status, and liver fibrosis in patients with non-alcoholic fatty liver (NAFLD): a study protocol for a randomized controlled clinical trial

**DOI:** 10.1186/s40795-023-00794-x

**Published:** 2023-12-08

**Authors:** Mehdi Karimi, Maryam Mofidi Nejad, Camellia Akhgarjand, Amir Ali Sohrabpour, Hossein Poustchi, Hossein Imani, Hamed Mohammadi

**Affiliations:** 1 Department of Clinical Nutrition, School of Nutritional Sciences and Dietetics, Tehran University of Medical Sciences, P.O. Box 14155-6117, Tehran, Iran; 2 Department of Community Nutrition, School of Nutritional Sciences and Dietetics, Tehran University of Medical Sciences, Tehran, Iran; 3 Tehran Gastroenterology and Hepatology Centre (Masoud Clinic), Tehran, Iran; 4 Liver and Pancreatobiliary Diseases Research Center, Digestive Diseases Research Institute, Tehran University of Medical Sciences, Tehran, Iran

**Keywords:** Intermittent fasting diet, Low-calorie diet, Non-alcoholic fatty liver, Randomized controlled clinical trial

## Abstract

**Introduction:**

Non-alcoholic fatty liver disease (NAFLD) is a common liver disease characterized by an increase in fat in liver cells. The outbreak of NAFLD is estimated to be 32.4% worldwide, with higher rates in Asia and Iran. Nutritional factors such as excessive calorie intake, high fructose intake, copper deficiency, and increased iron intake play an important role in NAFLD. Since there is no specific treatment for NAFLD, intermittent fasting (IF) diet has been suggested as an alternative treatment for obesity and related complications. Previous studies showed the potential positive effects of IF on metabolic health and the reduction of oxidative stress in NAFLD. This randomized controlled trial (RCT) will be aimed to examine the effect of the IF diet in comparison with a low-calorie diet (LCD) on lipid profile, glycemic status, and liver fibrosis in patients with NAFLD.

**Methods and analysis:**

This is a parallel randomized clinical trial conducted on 52 overweight and obese patients with NAFLD. Participants will be randomly assigned to receive either 16:8 IF (fasting from 8 P.M. to 12 P.M. the next day) or a low-calorie (55% carbohydrate- 30% fat, 15% protein) diet for 12 weeks. Anthropometric measurements, liver assessments, and metabolic evaluations will be assessed before and after the intervention. Primary outcomes include liver steatosis and fibrosis, while secondary outcomes include liver function enzymes, insulin resistance, lipid profile, and anthropometric measurements.

**Discussion:**

Since obesity and insulin resistance are the most important risk factors of NAFLD, and there is no treatment for it, it seems that lifestyle changes such as low caloric diet like IF and exercise can improve lipid metabolism and liver enzymes.

**Trial registration:**

Iranian registry of clinical trials (IRCT20170202032367N5).

## Introduction

Nonalcoholic fatty liver disease (NAFLD) is one of the most common liver diseases [1, 2] with a variety of conditions including simple steatosis, nonalcoholic steatohepatitis (NASH), and fibrosis [3, 4]. NAFLD is characterized by an increase in the amount of fat within liver cells without excessive alcohol consumption [5]. The prevalence of NAFLD was estimated to be 32.4% worldwide [6] and in Asia, the outbreak of NAFLD is reported to be 29.6% [7]. In addition, in the Iranian population, the prevalence is estimated to be around 33.9% [8]. Diet appears to play an important role in improving NAFLD [9]. Dietary factors involved in this disease include excess calorie intake, amount of carbohydrates consumed, types of fats consumed, high fructose intake, copper deficiency, and increased iron intake [10]. Currently, there is no specific treatment for NAFLD, however, a combination of increased physical activity and nutritional modifications are the best alternative treatments for this disease [11, 12]. Adherence to a variety of diets, including low-fat and low-carbohydrate diets, has been less successful in reducing and maintaining weight loss and improving metabolic risk factors in obese individuals, so preventing or controlling associated risk factors can be considered as an alternative treatment [13–15]. Recently, intermittent fasting (IF) diet has been used to limit daily energy intake to treat obesity and its complications, and as an anti-aging method to extend lifespan compared to traditional low-calorie diets (LCDs( [16, 17]. Several studies suggest that compared to LCD, IF may improve metabolic health including significant reductions in insulin resistance, blood pressure, and blood lipids [18]. Some studies have shown that weight gain is associated with increased inflammation and oxidative stress, which may be one of the triggers of NAFLD [19]. On the other hand, one of the positive effects of the IF diet on reducing oxidative stress was the change in body weight and slight upregulation of the sirtuin-3 gene (SIRT3), which is one of the effective therapeutic strategies in fatty liver [20, 21].

Considering the high complications of this disease and the direct and indirect cost of its complications, it seems that low-cost and effective methods such as IF diets can be effective in controlling and preventing the progression of this disease. Therefore, we decided to investigate the effects of an IF diet on lipid profile, blood glucose, and liver fibrosis markers in patients with NAFLD as a novel disease progression prevention and management method that has been studied to a limited extent [22, 23].

### Methods and analysis

The present study is a parallel randomized controlled clinical trial that will be performed at Masoud Clinic in Tehran, Iran on patients with NAFLD. All patients must complete and sign an informed written consent before completing their registration. The research has been approved by the Bioethics Committee of TUMS, Tehran, Iran (No. IR.TUMS.MEDICINE.REC.1400.682). This RCT was registered on the IRCT web page (IRCT20170202032367N5).

### Participants

Participants with NAFLD will be recruited based on the inclusion criteria. NAFLD will be diagnosed according to the Fibro Scan (Metavir-score ≤ F2 and controlled attenuation parameter (Cap) score > 263). To better reflect population diversity and obtain more generalizable results, individuals will be recruited through popular online advertising applications.

### Inclusion criteria

This clinical trial will be performed on both men and women with NAFLD (Metavir-score ≤ F2 and controlled attenuation parameter (Cap) score > 263), BMI between 25 to 35 kg/m^2^, and aged from 20 to 50 years.

### Exclusion criteria

Patients with the following criteria will be excluded from the clinical trial: pregnancy or breastfeeding, consumption of alcohol, smoking or drug use, suffering from other liver diseases, diabetes, taking hepatotoxic (phenytoin, lithium, tamoxifen, antibiotics), antihyperglycemic, weight loss medications, corticosteroids and persons performed night-shift work, and following a special diet in the past three months.

### Sample size calculation

To compare the studied variables between the two groups, considering the error of 5%, the power of 80%, and the effect size of 0.8 (based on Cohen's criterion), the minimum sample size is equal to 26 people in each group and a total of 52 people will be determined.

*t tests—Means*: Difference between two independent means (two groups)

*Analysis:* A priori: Compute required sample size

*Input:* Tail(s) = Two

Effect size d = 0.80

α err prob = 0.05

Power (1-β err prob) = 0.8

Allocation ratio N2/N1 = 1

*Output*

Noncentrality parameter δ = 2.8844410

Critical t = 2.0085591

Df = 50

Sample size group 1 = 26

Sample size group 2 = 26

Total sample size = 52

Actual power = 0.8074866



### Study design

**Fig. 1:**
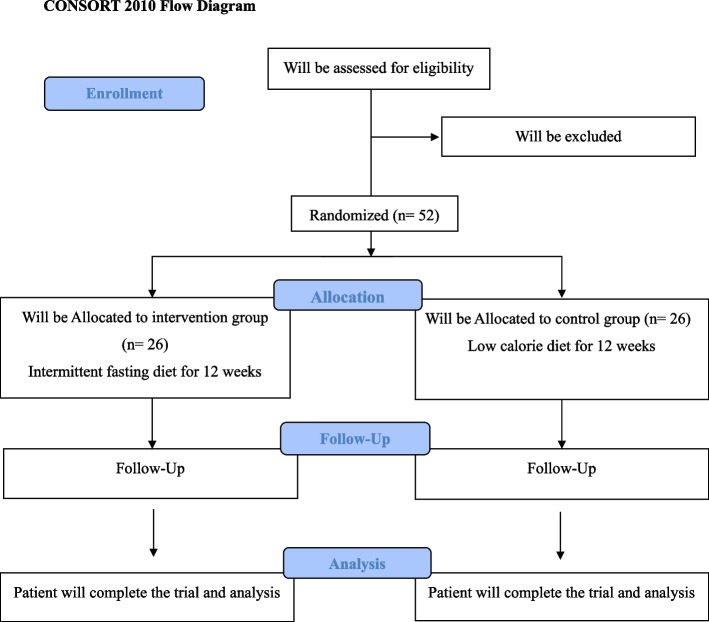
Diagram of the study design

**Fig. 2:**
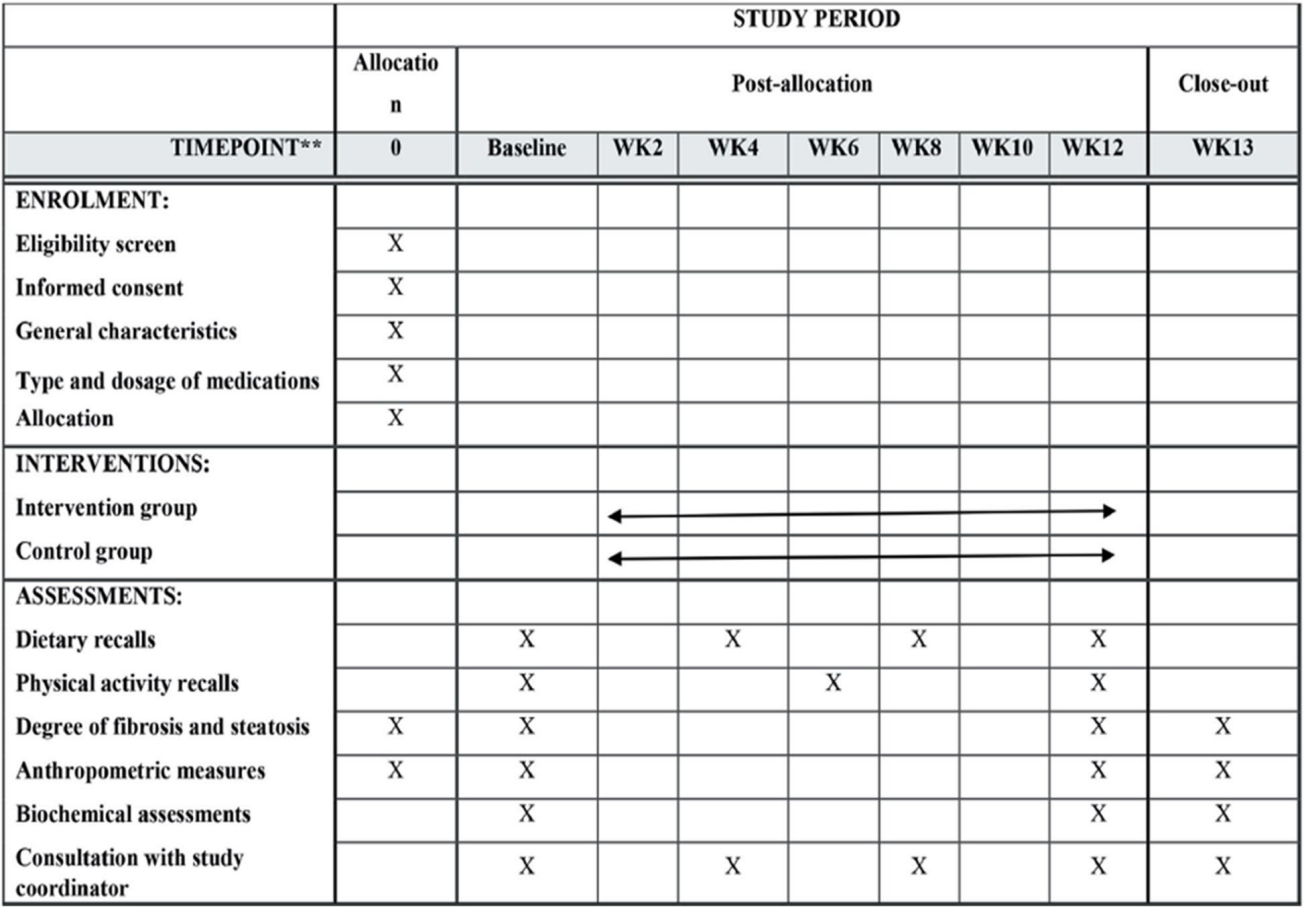
Flow chart of enrolment, intervention, and assessments. The ‘X’ refers to the time of allocation, intervention, and assessment of variables. NAFLD, non-alcohol fatty liver disease

A diagram of the study design is demonstrated in Fig. 1. A flow chart of the study process is demonstrated in Fig. 2. A total of 52 patients with NAFLD will be screened based on the inclusion criteria. After participant recruitment from online advertisements and social media, 52 participants who meet the eligibility criteria will be enrolled for the trial. We used stratified block randomization with a block size of 2 Patients will be randomly assigned to either an intervention or control group based on BMI (25–30 and 30–35) and gender (male or female). First, we select patients who meet the criteria. Then that variable is used to match the next person with the first person. After all, two patients with similar characteristics are in the same block. Finally, two subjects in the same block are randomly assigned to either the IF diet or the low-calorie diet using random assignment software. All individuals who meet the inclusion criteria and voluntarily participate in the study will receive complete information regarding the purpose of the study, the type of intervention, and the duration of the study. Then, written informed consent will be obtained from individuals. The current clinical trial will be done based on the Helsinki Declaration. All participants will be informed that all services will be free of charge and that they can withdraw from the study at any time. Each person will be given a pre-made meal plan booklet with a set of menus and recipes for breakfast, lunch, dinner, and snacks based on the recommended diet.

### Intervention

Participants will be randomly assigned to either intervention or control group. During the conversation with the patients, each patient will be assigned to either the IF diet or the LCD. Individuals in the intervention group will follow a 16:8 IF diet that will allow them to consume only water and non-energy beverages such as tea, coffee, and sugar-free chewing gum for 16 h and eat food ad libitum for 8 h. The control group will receive LCD with 3 main dishes and 3 snacks with 55% carbohydrate, 30% fat, and 15% protein (300 kcal less than energy requirements) for 12 weeks.

### Compliance

Participants' food intake will be recorded every week to assess how well they are adhering to their diet. Participants will complete a total of 12 food records. Patients will be requested to report their dietary intakes using home measurements to complete their daily recall. Finally, home measurements will be converted to grams using the brochure provided. Participants' total food intake will be examined using the average of all meal recalls during the intervention period. We will calculate energy intake, micronutrients, and macronutrients from dietary recalls using Nutrition IV software (First Databank, San Bruno, CA, USA) modified for Iranian meals. Text messages will be sent to patients to increase diet compliance and remind them to follow the diet.

### Intervention safety

There are no important side effects associated with fasting diets. To assess possible side effects, the participant will be asked to report any changes during the trial with an assessment of their diet via telephone interview.

### Assessment of variables

#### Primary and secondary outcome

The primary outcome of this RCT will be determining the level of steatosis and liver fibrosis stage by FibroScan. The major secondary outcome variables will be the measurement of serum liver function enzymes including aspartate aminotransferase (AST), alanine aminotransferase (ALT), and gamma-glutamyl transferase (GGT), and fasting blood insulin (FBI), fasting blood sugar (FBS), homeostatic model assessment for insulin resistance (HOMA-IR), quantitative insulin sensitivity check index (QUICKI), lipid profile including serum total cholesterol (TC), serum triglycerides (TG), low-density lipoprotein cholesterol (LDL_C), high-density lipoprotein cholesterol (HDL_C) and anthropometric indicators including weight, height, and waist circumference (WC).

#### Assessment of dietary intake

As mentioned above, dietary intake will be assessed using 24-h dietary recall (including one weekend day and two working days) at baseline, weeks four, eight, and twelve of the intervention. Experienced and trained professionals will collect these recalls from the participants, and the mean intake of food items during these four recalls will be calculated in grams per day. The nutrient intake of individuals will be calculated using N4 software (Nutritionist4).

#### Assessment of hepatic fibrosis and steatosis

Steatosis and fibrosis of the liver will be assessed in study participants using transient elastography and CAP, respectively with FibroScan before and after intervention by the same gastroenterologist. FibroScan is a noninvasive diagnostic ultrasound-based device used to measure liver scarring or fibrosis. To assess liver stiffness, transient elastography with fibroscan will be performed. Patients are requested to fast for at least 2 h before the test. Patients will be laid in the dorsal decubitus position and measurements will be taken with a probe placed on the intercostal space and right lobe of the liver. FibroScan takes 10 min to complete and liver stiffness is expressed in kilopascals (kPa) [24]:

- F0/F1 (kPa < 7.3): No fibrosis or mild fibrosis
- F2 (kPa 7.3–12.5): Moderate fibrosis
- F3 (kPa 12.6–17.6): Severe fibrosis

- F4 (kPa > 17.6): Cirrhosis

Also, we will use the FibroScan device's CAP test to assess steatosis of the liver. Individuals will be classified based on CAP cutoffs as follows [25]:

- S0: no steatosis: CAP < 238
- S1: mild steatosis: 238 ≤ CAP < 260
- S2: moderate steatosis: 260 ≤ CAP < 293
- S3: severe steatosis: 293 ≤ CAP

#### Biochemical assessments

Biochemical factors will be assessed in the TUMS nutrition faculty laboratory. To measure liver enzyme levels (ALT, AST, and GGT), blood glucose, and serum lipids (including TC, serum TG, and HDL_C), a 10 mL sample of venous blood will be collected after 10–12 h fast. After 30 min in the laboratory environment to clot the blood, it will be centrifuged at 3000 rpm for 10 min to extract serum. ALT, AST enzyme levels, and serum lipid profile, including TC, HDL-C, LDL-C, and serum TG, will be measured using commercial kits and enzymatic methods (Pars Azmoon kit, Tehran, Iran). The GGT level will also be measured using the International Federation of Clinical Chemistry (IFCC) recommended calorimetric-kinetic method. Blood glucose will be measured using the enzymatic glucose oxidase method using a glucose-specific kit. Also, a specific kit using the ELISA method will be used to measure FBI, and HOMA-IR and QUICKI indices will be calculated based on the formula below using FBI and fasting blood glucose (FBG) measurements [26, 27]:

$$\text{QUICKI}=1/\left(\log\;(\text{fasting insulin}\; \upmu\text{U}/\text{mL}\right)+\log\;\left(\text{fasting}\;\text{glucose mg}/\text{dL}\right)$$

$$\text{HOMA1-IR} = \left(\text{fasting}\;\text{insulin}\;\left(\upmu\mathrm{U}/\text{L}\right)\times\text{fasting}\;\text{glucose}\;\left(\text{mmol}/\text{L}\right)\right)\!/22.5$$

#### Anthropometric measures

At the beginning and end of the study, data will be collected regarding anthropometric measurements, including height, weight, BMI, and WC. Body weight will be measured in a fasted state, wearing minimal clothing and without shoes, using a digital scale with a precision of 100 g. WC will be measured using a measuring tape, measuring the distance between the suprailiac bone and the last rib with a precision of 0.5 cm. Standing height will be measured using a standard stadiometer without shoes, with a precision of 0.5 cm. BMI will be calculated using the measured height and weight (weight in kilograms/height in square meters) [28].

### Statistical analysis

Statistical data will be analyzed using SPSS software version 22. The results will be reported as mean ± standard deviation (SD). The normality of variable distributions will be checked using the Kolmogorov–Smirnov test. If a variable does not follow a normal distribution, a logarithmic transformation will be applied. The independent t-test will be used to compare the consumption of food and nutrients between the two groups, and the chi-square test will be used to compare qualitative variables between the two groups. The analyses will be performed based on the intention-to-treat (ITT) approach. Missing values will be treated according to the linear regression method. ANCOVA with adjustment with baseline values will be used to assess the effect of intervention on dependent variables. *p*-value < 0.05 will be considered statistically significant.

## Discussion

Obesity resulting from excessive calorie intake is a major risk factor for insulin resistance (IR), which is a key factor in the development of metabolic syndrome and type 2 diabetes [29]. IR promotes the development of NAFLD in a subgroup of patients, which can progress to a more severe form, namely NASH [30]. Despite weight loss, there is still no consensus on appropriate strategies for managing NAFLD patients, and unfortunately, our knowledge of more effective treatment options to minimize or its removal remains limited [31, 32]. Many guidelines support recommendations on risk factor control and lifestyle changes, including diet and physical activity [33]. Furthermore, for the treatment of obesity and metabolic parameters, energy restriction can be considered as the main mechanism [34]. Energy restriction in an IF diet can lead to the mobilization of free fatty acids, increased fat oxidation, and thus the generation of ketone bodies [22]. Despite this, a study conducted by Santos HO and colleagues showed that IF has beneficial effects on lipid metabolism at the molecular level [35]. By reducing apolipoprotein B (apoB) production, increasing fatty acid oxidation, and reducing TG content in the liver, IF may help improve lipid profiles and reduce the risk of metabolic diseases [35]. On the other hand, because NAFLD and atherosclerotic dyslipidemia are related to each other [36], recent studies have shown that IF leads to a decrease in the levels of very low-density lipoprotein cholesterol (VLDL_C), LDL_C, and small dense low-density lipoprotein cholesterol (sdLDL_C), highlighting the anti-atherogenic effects of IF diets [35, 37]. In individuals, moreover, with NAFLD, liver function enzymes (AST, ALT, and GGT) are commonly considered in clinical assessment. Johari and colleagues after 8 weeks of calorie restriction, observed a reduction in AST and ALT levels in patients with NAFLD [33]. The authors described this reduction in liver tests as an improvement in steatosis or visceral fat in the liver [33].

The results of this study can be applied to the management of NAFLD patients. Because dietary modification is likely the most cost-effective method for disease management and treatment, the results of our study can be widely used by hepatologists and nutritionists.

In conclusion, because dietary modification is most likely a cost-effective method of disease management and treatment, the results of this study can be applied to the management of NAFLD patients.

### Strengths and limitations

This is the first clinical trial to assess the effects of the 16:8 IF diet in comparison with LCD on lipid profile, glycemic status, and liver fibrosis in patients with NAFLD. It should be noted that this procedure is inexpensive and the patient will not incur additional costs. We will use stratified block randomization to match participants based on several confounding variables that may affect the results. People, moreover, will participate via public announcements. Therefore, all participants can be eager to comply with dietary recommendations. Some limitations need to be considered. First, we will assess adherence to the IF diet through self-reported food recalls. Therefore, self-reporting of food consumption may influence study results. Second, although FibroScan has been established as a noninvasive and reliable method for diagnosing fibrosis and steatosis in NAFLD patients, the gold standard for assessing liver steatosis is magnetic resonance imaging-proton density fat fraction (MRI-PDFF). Third, we will assess physical activity using a physical activity questionnaire. Fourth, stool sample measurements will not be performed in this study. Therefore, dysbiosis of intestinal flora cannot be measured. Eventually, we have no access to bio-electrical impedance analysis (BIA) to examine body composition in this study.

## Data Availability

Data generated or analyzed during the current study will be available from the corresponding author upon reasonable request.

## References

[1] Rinella ME (2015). Nonalcoholic fatty liver disease: a systematic review. JAMA.

[2] Vernon G, Baranova A, Younossi ZM (2011). Systematic review: the epidemiology and natural history of non-alcoholic fatty liver disease and non-alcoholic steatohepatitis in adults. Aliment Pharmacol Ther.

[3] Anstee QM, Targher G, Day CP (2013). Progression of NAFLD to diabetes mellitus, cardiovascular disease or cirrhosis. Nat Rev Gastroenterol Hepatol.

[4] Gaggini M, Morelli M, Buzzigoli E, DeFronzo RA, Bugianesi E, Gastaldelli A (2013). Non-alcoholic fatty liver disease (NAFLD) and its connection with insulin resistance, dyslipidemia, atherosclerosis and coronary heart disease. Nutrients.

[5] Elshaghabee FM, Rokana N, Panwar H, Heller KJ, Schrezenmeir J (2019). Probiotics as a dietary intervention for reducing the risk of nonalcoholic fatty liver disease. Pharmaceuticals from Microbes: Impact on Drug Discovery.

[6] Riazi K, Azhari H, Charette JH, Underwood FE, King JA, Afshar EE (2022). The prevalence and incidence of NAFLD worldwide: a systematic review and meta-analysis. Lancet Gastroenterol Hepatol.

[7] Wong S-W, Chan W-K (2020). Epidemiology of non-alcoholic fatty liver disease in Asia. Indian J Gastroenterol.

[8] Moghaddasifar I, Lankarani K, Moosazadeh M, Afshari M, Ghaemi A, Aliramezany M (2016). Prevalence of non-alcoholic fatty liver disease and its related factors in Iran. Int J Organ Transplant Med.

[9] Karlas T, Wiegand J, Berg T (2013). Gastrointestinal complications of obesity: non-alcoholic fatty liver disease (NAFLD) and its sequelae. Best Pract Res Clin Endocrinol Metab.

[10] Worm N (2020). Beyond body weight-loss: dietary strategies targeting intrahepatic fat in NAFLD. Nutrients..

[11] Finer N (2022). Weight loss interventions and nonalcoholic fatty liver disease: optimizing liver outcomes. Diabetes Obes Metabolism.

[12] Domingues I, Leclercq IA, Beloqui A (2023). Nonalcoholic fatty liver disease: current therapies and future perspectives in drug delivery. J Control Release.

[13] Patel AA, Torres DM, Harrison SA (2009). Effect of weight loss on nonalcoholic fatty liver disease. J Clin Gastroenterol.

[14] Hu T, Mills KT, Yao L, Demanelis K, Eloustaz M, Yancy WS (2012). Effects of low-carbohydrate diets versus low-fat diets on metabolic risk factors: a meta-analysis of randomized controlled clinical trials. Am J Epidemiol.

[15] Hansen CD, Gram-Kampmann E-M, Hansen JK, Hugger MB, Madsen BS, Jensen JM (2023). Effect of calorie-unrestricted low-carbohydrate, high-fat diet versus high-carbohydrate, low-fat diet on type 2 diabetes and nonalcoholic fatty liver disease: a randomized controlled trial. Ann Intern Med.

[16] Mattson MP, de Cabo R (2020). Effects of intermittent fasting on health, aging, and disease. Reply. N Engl J Med.

[17] Patterson RE, Sears DD (2017). Metabolic effects of intermittent fasting. Annu Rev Nutr.

[18] Liu K, Liu B, Heilbronn LK (2020). Intermittent fasting: what questions should we be asking?. Physiol Behav..

[19] Ashraf N, Sheikh T (2015). Endoplasmic reticulum stress and oxidative stress in the pathogenesis of non-alcoholic fatty liver disease. Free Radic Res.

[20] Faris ME, Hussein RN, Al-Kurd RA, Al-Fararjeh MA, Bustanji YK, Mohammad MK (2012). Impact of Ramadan intermittent fasting on oxidative stress measured by urinary 15–isoprostane. J Nutr Metab.

[21] Wegman MP, Guo MH, Bennion DM, Shankar MN, Chrzanowski SM, Goldberg LA (2015). Practicality of intermittent fasting in humans and its effect on oxidative stress and genes related to aging and metabolism. Rejuven Res.

[22] Kord Varkaneh H, Salehi Sahlabadi A, Găman MA, Rajabnia M, Sedanur Macit-Çelebi M, Santos HO (2022). Effects of the 5:2 intermittent fasting diet on non-alcoholic fatty liver disease: a randomized controlled trial. Front Nutr..

[23] Yin C, Li Z, Xiang Y, Peng H, Yang P, Yuan S (2021). Effect of intermittent fasting on non-alcoholic fatty liver disease: systematic review and meta-analysis. Front Nutr..

[24] Mohammed MN, Al-Nadry MH, Abdel-halim MM, Abdelaziz AO, Lithy R, Abdelmaksoud AH, Shousha HI. Role of Transient Elastography (Fibroscan) in Early prediction of Hepatitis C Virus Related Hepatocellular Carcinoma. Al-Azhar International Medical Journal. 2023.

[25] Sansom SE, Martin J, Adeyemi O, Burke K, Winston C, Markham S (2019). Steatosis rates by liver biopsy and transient elastography with controlled attenuation parameter in clinical experience of hepatitis C virus (HCV) and human immunodeficiency virus/HCV coinfection in a large US hepatitis clinic. Open Forum Infect Dis.

[26] Pour Abbasi MS, Shojaei N, Farhangi MA (2022). Low-carbohydrate diet score is associated with improved blood pressure and cardio‐metabolic risk factors among obese adults. Physiological Rep.

[27] Mokhtari B, Abdoli-Shadbad M, Alihemmati A, Javadi A, Badalzadeh R (2022). Alpha-lipoic acid preconditioning plus ischemic postconditioning provides additional protection against myocardial reperfusion injury of diabetic rats: modulation of autophagy and mitochondrial function. Mol Biol Rep.

[28] Khanna D, Peltzer C, Kahar P, Parmar MS (2022). Body mass index (BMI): a screening tool analysis. Cureus.

[29] Fukunaka A, Fujitani Y (2018). Role of zinc homeostasis in the pathogenesis of diabetes and obesity. Int J Mol Sci..

[30] Yin H, Shi A, Wu J (2022). Platelet-activating factor promotes the development of non-alcoholic fatty liver disease. Diabetes Metab Syndr Obes.

[31] Dashti F, Alavian SM, Sohrabpour AA, Mousavi SE, Keshavarz S-A, Esmaillzadeh A (2023). Protocol: Effect of a moderately carbohydrate-restricted diet on liver enzymes, steatosis and fibrosis in normal-weight individuals with non-alcoholic fatty liver disease: study protocol for a parallel randomised controlled clinical trial. BMJ Open.

[32] Tarantino G, Citro V, Capone D (2019). Nonalcoholic fatty liver disease: a challenge from mechanisms to therapy. J Clin Med..

[33] Johari MI, Yusoff K, Haron J, Nadarajan C, Ibrahim KN, Wong MS (2019). A randomised controlled trial on the effectiveness and adherence of modified alternate-day calorie restriction in improving activity of non-alcoholic fatty liver disease. Sci Rep.

[34] Casanova N, Beaulieu K, Oustric P, O’Connor D, Gibbons C, Blundell JE (2023). Increases in physical activity are associated with a faster rate of weight loss during dietary energy restriction in women with overweight and obesity. Br J Nutr.

[35] Santos HO, Macedo RC (2018). Impact of intermittent fasting on the lipid profile: Assessment associated with diet and weight loss. Clin Nutr ESPEN.

[36] Akhtar DH, Iqbal U, Vazquez-Montesino LM, Dennis BB, Ahmed A (2019). Pathogenesis of insulin resistance and atherogenic dyslipidemia in nonalcoholic fatty liver disease. J Clin Transl Hepatol.

[37] Laddu DR, Lavie CJ, Phillips SA, Arena R (2021). Physical activity for immunity protection: inoculating populations with healthy living medicine in preparation for the next pandemic. Prog Cardiovasc Dis.

